# Complete Genome Sequences of Three Campylobacter jejuni Phage-Propagating Strains

**DOI:** 10.1128/genomeA.00514-18

**Published:** 2018-06-14

**Authors:** J. C. Sacher, E. Yee, C. M. Szymanski, W. G. Miller

**Affiliations:** a Department of Biological Sciences, University of Alberta, Edmonton, Alberta, Canada; b Produce Safety and Microbiology Research Unit, Agricultural Research Service, U.S. Department of Agriculture, Albany, California, USA; c Department of Microbiology and Complex Carbohydrate Research Center, University of Georgia, Athens, Georgia, USA

## Abstract

Bacteriophage therapy can potentially reduce Campylobacter jejuni numbers in livestock, but it requires a detailed understanding of phage-host interactions. C. jejuni strains readily infected by certain phages are designated as phage-propagating strains. Here, we report the complete genome sequences of three such strains, NCTC 12660, NCTC 12661, and NCTC 12664.

## GENOME ANNOUNCEMENT

Campylobacter jejuni causes diarrheal disease worldwide, and C. jejuni infections arise from consuming and mishandling contaminated poultry (1–3). Phages are being explored as antibiotic alternatives to reduce this burden (4–6). Phages are highly strain specific, so understanding the factors that contribute to this specificity, including capsular polysaccharides (CPSs), flagella (7), and restriction/modification systems (8, 9), can maximize the strain range targeted (10).

C. jejuni strains were historically tracked based on phage susceptibility (11, 12). For these typing schemes, each phage was designated a readily infected “phage-propagating” strain. To identify factors governing phage susceptibility in C. jejuni, we sequenced the genomes of three C. jejuni phage-propagating strains isolated from chickens (12), NCTC 12660, NCTC 12661, and NCTC 12664.

Whole-genome sequencing was performed using the PacBio RS and Illumina MiSeq sequencing platforms. PacBio sequence data were assembled to construct a single closed chromosomal contig for each strain. MiSeq reads were used to validate base calls and to determine the variability at each poly-G tract. Protein-, rRNA-, and tRNA-coding genes were identified as described previously (13). The genome sizes ranged from 1.61 to 1.68 Mb with an average GC content of 30.6%. The three genomes show high similarity to strain NCTC 11168, although NCTC 12660 has at least one small inversion compared to NCTC 11168. These four genomes encode a similar number of genes and pseudogenes, with the genome of NCTC 12660 slightly larger due to the presence of a genomic island. Many of the pseudogenes identified were conserved across all or most of the three strains and NCTC 11168.

We identified several differences in restriction/modification (R/M) and clustered regularly interspaced short palindromic repeats (CRISPR)/CRISPR-associated (Cas) systems between these strains. Relative to the others, NCTC 12661 lacks a type I R/M system, NCTC 12661 and NCTC 12664 lack the type IIG restriction endonuclease (RE) *cj1051*, NCTC 12661 uniquely encodes a type III R/M system and the type IIG RE (locus tag CJ12661_0039), and the type IV R/M system subunit *mcrB* is a pseudogene in NCTC 12660. Interestingly, all but NCTC 12664 encode a full type II-C CRISPR/Cas system, with *cas9* a pseudogene in NCTC 12664.

CPS variability influences C. jejuni phage susceptibility (7, 14), but flagellar glycans play an unknown role (15). Strains NCTC 12661 and NCTC 12664 cluster separately from NCTC 12660 and NCTC 11168 in CPS and flagellar glycosylation gene content, which could lead to differences in phage-host interactions. In addition to C. jejuni strain-strain variation, within-strain genome variation has been observed (16, 17). We compared our NCTC 12661 sequence to two prior genomes sequenced for this strain: GenBank accession numbers CP010906 (18) and CP020045 (17). Two alleles for *pseD*, encoding the flagellar acetamidino-substituted pseudaminic acid transferase, were previously observed (17, 19). Our NCTC 12661 *pseD* was 100% and 86% identical to these alleles. The *pseD* sequence from the earliest NCTC 12661 genome (18) has regions of similarity to each of the *pseD* genes from the subsequently sequenced genomes. This suggests possible recombination, although sequencing or assembly issues could be responsible. Either scenario could be explained by the many *pseD* homologs encoded by most C. jejuni strains (20). This example highlights the plasticity of C. jejuni genomes.

### Accession number(s).

The complete genome sequences of C. jejuni strains NCTC 12660, NCTC 12661, and NCTC 12664 have been deposited in GenBank under the accession numbers CP028910, CP028911, and CP028912, respectively.
